# CNN-based diagnosis models for canine ulcerative keratitis

**DOI:** 10.1038/s41598-019-50437-0

**Published:** 2019-10-02

**Authors:** Joon Young Kim, Ha Eun Lee, Yeon Hyung Choi, Suk Jun Lee, Jong Soo Jeon

**Affiliations:** 1 Veterinary Medical Teaching Hospital, Konkuk University, Seoul, 05029 Republic of Korea; 2 Division of Business Administration, College of Business, Kwangwoon University, Seoul, 01897 Republic of Korea

**Keywords:** Animal physiology, Mechanical engineering

## Abstract

The purpose of this methodological study was to develop a convolutional neural network (CNN), which is a recently developed deep-learning-based image recognition method, to determine corneal ulcer severity in dogs. The CNN model was trained with images for which corneal ulcer severity (normal, superficial, and deep) were previously classified by veterinary ophthalmologists’ diagnostic evaluations of corneal photographs from patients who visited the Veterinary Medical Teaching Hospital (VMTH) at Konkuk University and 3 different veterinary ophthalmology specialty hospitals in Korea. The original images (depicting normal corneas (36) and corneas with superficial (47) ulcers, deep (47) ulcers), flipped images (total 520), rotated images (total 520), and both flipped and rotated images (total 1,040) were labeled, learned and evaluated with GoogLeNet, ResNet, and VGGNet models, and the severity of each corneal ulcer image was determined. To accomplish this task, models based on TensorFlow, an open-source software library developed by Google, were used, and the labeled images were converted into TensorFlow record (TFRecord) format. The models were fine-tuned using a CNN model trained on the ImageNet dataset and then used to predict severity. Most of the models achieved accuracies of over 90% when classifying superficial and deep corneal ulcers, and ResNet and VGGNet achieved accuracies over 90% for classifying normal corneas, corneas with superficial ulcers, and corneas with deep ulcers. This study proposes a method to effectively determine corneal ulcer severity in dogs by using a CNN and concludes that multiple image classification models can be used in the veterinary field.

## Introduction

Deep learning diagnostic tools for image recognition have recently been tested in many medical fields. In the field of imaging diagnostics, the use of such tools have been reported in The Veterinary Journal^1,2^. Recent advances in computer hardware technology, such as high performance graphic processing units (GPUs), have permitted the development of deep neural networks (DNNs). Deep learning algorithms are an evolution of neural networks and are currently used in a variety of medical and industrial applications^3,4^.

**Figure 1:**
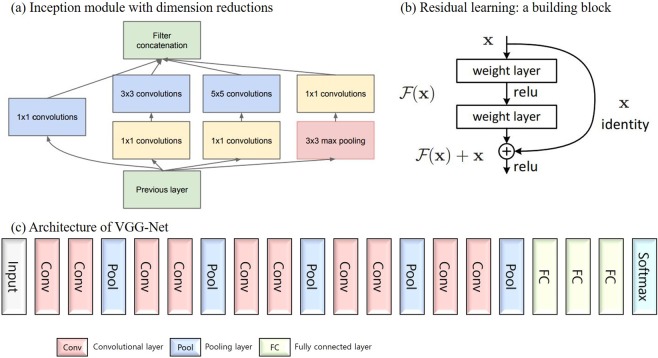
Architectures of GoogLeNet. (**a**) Inception module with dimension reduction, ResNet. (**b**) Residual learning: a building block, and VGGNet. (**c**) Architecture of VGGNet.

Deep learning fundamentally consists of a deep neural network structure with several layers. An artificial neural network based on the backpropagation^5^ algorithm was highly anticipated in the 1990s; such a network would utilize logic that corrects the error of each neuron after analyzing the error in the reverse direction at the output side when the error occurs. However, research has stagnated because learning in artificial neural network models becomes more difficult as the number of layers increases. Since the mid-2000s, artificial neural networks have been improved with respect to learning methods: huge amounts of data have been made available, and the hardware environment has been improved, leading to remarkable performance improvements. In addition, the dataset overfitting problem, which was a persistent issue with artificial neural networks, was improved by using the dropout method^6^, and generalizability was improved^7^. Among deep learning algorithms, the convolutional neural network (CNN) became prominent based on its use in handwriting recognition in the late 1990s. Because a CNN is constructed to essentially imitate human visual processing, it is thought to be suitable for image processing. Recent advances in technology have led to an increased model hierarchy and significant performance improvements. In particular, since the development of CNNs that achieve good performances on image recognition tasks, great progress has been achieved in learning image data. This progress has allowed the models to extract features that can be abstracted and displayed well. A CNN consists of convolutional, subsampling, and fully connected layers. A convolutional layer extracts image characteristics, and a subsampling layer provides downsampling and location invariance features for the image. By repeating these two layers, high-dimensional information can be extracted from the image effectively. Representative CNN models include GoogLeNet^8^, ResNet^9^ and VGGNet^10^. GoogLeNet uses Inception modules, which were introduced to more effectively represent the local characteristics of a space (Fig. 1(a)). Kernels of 1 × 1, 3 × 3, and 5 × 5 segment, separate, and calculate the local space characteristics and the last layer of the Inception module combines them all. The 1 × 1 convergence layer is connected and used to reduce the cost of computing the 3 × 3 and 5 × 5 convolutional layers inside the Inception module. The ReLU activation function is used in the 1 × 1 convolutional layer to reduce the complexity of calculating the 3 × 3 and 5 × 5 convolutional layers. GoogLeNet uses nine repeated Inception modules. The ResNet model forms a deep network using residual blocks (Fig. 1(b)). The residual blocks used for learning preserve the features of the input data through identity skip connections. ResNet overcomes some of the problems of a deep network, such as the vanishing gradient problem, and it reduces the complexity. ResNet mostly uses 3 × 3 filters, similar to VGGNet, and the output feature map applies the same number of filters (or doubles the number of filters if the feature map is halved). VGGNet is an effective model containing multiple filters that have small acceptance fields to increase the network size (Fig. 1(c)). Instead of filters with large receptive fields, the smallest filter (i.e., the 3 × 3 filter) is repeatedly applied to increase the number of layers, which has been found to be advantageous. VGGNet uses small, 3 × 3 receptive fields throughout the whole network. A stack of two 3 × 3 filters has an effective receptive field of 5 × 5, while three such layers have a 7 × 7 effective receptive field. These three models were used in this study.

Ulcerative keratitis is an extremely common veterinary ophthalmic disease in dogs^11^. A corneal ulcer is present when a break occurs in the corneal epithelium that exposes the underlying corneal stroma^12^. Clinically, this results in lacrimation, blepharospasm, photophobia, conjunctival hyperemia, corneal edema, and possibly miosis and aqueous flare^12^. Fortunately, corneal ulcers are considered a treatable disease relative to other ophthalmic diseases that threaten dog vision. However, complicated deep ulcers, such as those with microbial infection, may lead to impaired vision due to corneal scarring or—when corneal perforation occurs—to anterior synechia formation^12^. Severe ulcerative keratitis may lead to eye loss due to endophthalmitis, glaucoma, phthisis bulbi, or a combination^12^. Corneal ulcers are classified by the depth of corneal involvement and by their underlying cause^12^. Thus, they are categorized as superficial corneal ulcers, stromal corneal ulcers, descemetoceles, and perforations depending on their depth. When dogs present with clinical signs of corneal ulcers, we can readily access useful treatment modalities^12^. Evaluating the severity of corneal ulcers is important for determining the best treatment method. However, general physicians often have difficulty classifying corneal ulcers. Furthermore, dog owners are often unaware of the severity of corneal ulcers, which can lead to delayed treatment. Therefore, the diagnosis of corneal ulcers using corneal images can allow dog owners to recognize the conditions of corneal ulcers and provide general physicians with diagnostic support. An ideal severity determination system should satisfy accuracy, repeatability, and ease of use requirements. As with all diseases, for ulcerative keratitis, it is necessary to have an assessment system that best reflects the clinical and epidemiological characteristics of dogs affected by different environmental factors. Although various studies exist on corneal ulcers^13,14^, no studies have used deep learning and/or other technical algorithms to assist in diagnosing such ulcers. Therefore, this study uses a CNN to determine the severity of corneal ulcers. Then, evaluations of the correct classification of the lesion images are performed to determine the utility of the CNN-based methods for diagnoses. In this methodological study, we first review the depth of corneal involvement and then evaluate the depth of corneal ulcerations to categorize the images as depicting normal corneas, corneas with superficial ulcers, or corneas with deep ulcers. In veterinary ophthalmology, clinically, physicians have tended to categorize corneal ulcers into four categories according to depth as superficial corneal ulcers, stromal corneal ulcers, descemetoceles, and perforations. However, it is difficult to prepare sufficient numbers of images of each category to adequately train a CNN model. Clinically, simple and superficial corneal ulcers are treated with topical or simple procedures, usually within 7 days of diagnosis. However, deep corneal ulcers, descemetocles, and corneal perforations often require surgery, while their medical treatments are similar to but more intensive than those used for simple ulcers. For this reason, in this study, deep corneal ulcers, descemetoceles and perforations were all classified as deep ulcers. Thus, we divided the images into three groups: normal, superficial ulcers, and deep ulcers.

## Results

Corneal images were obtained from 281 dogs. The breeds represented in the images were Shih-tzu (36%), Maltese (13%), Pekingese (12%), Poodle (8%), while together, other breeds represented the remaining 31% of the images. The images of superficial corneal ulcers (label 2) were obtained from Shih-tzu (34%), Poodle (13%), and Pekingese (12%) dogs, and the images of deep corneal ulcers (label 3) were obtained from Shih-tzu (51%), Pekingese (17%) and Maltese (11%) dogs. The mean age of all patients was 9.15 ± 4.3 (mean ± standard deviation (SD)) years. The mean age of the dogs with superficial corneal ulcers was 10.59 ± 3.7 (mean ± SD) years and the mean age of those with deep corneal ulcers was 7.81 ± 4.4 (mean ± SD). Overall, 35% of the dogs were castrated males, 14% were intact males, 26% were spayed females and 25% were intact females. The images of superficial corneal ulcers (label 2) were obtained from castrated males (31%), intact males (18%), spayed females (30%) and intact females (21%). The images of deep corneal ulcers (label 3) were from castrated males (33%), intact males (12%), spayed females (21%) and intact females (34%).

The maximum number of learning steps for each model was set to 10,000 to standardize the learning equity. Additionally, the RMSprop optimizer was used as an optimization strategy: the learning rate was set to 0.001, and the batch size was set to 32. The verification results of this experiment are shown in Tables 1 and 2. Inception_v2 reduced the amount of model computation through factorization and added auxiliary classifiers. Inception_v3 has the same structure as Inception_v2. It is adjusted to the optimal parameter value, and batch normalization (BN) is applied to the fully connected layer. Inception_v4 includes a process to reduce the image size by adding a reduction module to Inception_v3. ResNet_v2 differs from ResNet_v1 by applying the residual block method. Batch normalization and ReLU activation come before 2D convolution. The numbers after ResNet and VGGNet, indicate the number of layers. In classifying superficial and deep corneal ulcers, most models achieved accuracy rates of between 90% and 100%, and the performances of ResNet and VGGNet were better than that of GoogLeNet. In classifying normal corneas, corneas with superficial ulcers, and corneas with deep ulcers, most of the ResNet and VGGNet models achieved over 90% accuracy. The GoogLeNet models resulted in low accuracy relative to the other models. Additionally, the performance of rotated images showed low accuracy compared to the other images because rotation seems to generate artifacts in the images. Geometric operations such as rotation and scaling can destroy pixels and generate artifacts in the rotated images^15,16^.

**Table 1:** Accuracy (%) results of each model for predicting superficial and deep.

Models	Raw images			Flipped images			Rotated images			Flipped and rotated images		
	Superficial	Deep	Total	Superficial	Deep	Total	Superficial	Deep	Total	Superficial	Deep	Total
Inception_v1	100.0	100.0	100.0	100.0	92.3	96.0	100.0	92.3	96.0	100.0	84.6	92.0
Inception_v2	100.0	76.9	88.0	100.0	92.3	96.0	100.0	76.9	88.0	91.7	84.6	88.0
Inception_v3	100.0	92.3	96.0	100.0	100.0	100.0	100.0	92.3	96.0	91.7	84.6	88.0
Inception_v4	100.0	100.0	100.0	100.0	92.3	96.0	100.0	84.6	92.0	100.0	76.9	88.0
ResNet_v1_50	100.0	100.0	100.0	100.0	100.0	100.0	100.0	100.0	100.0	100.0	100.0	100.0
ResNet_v1_101	100.0	100.0	100.0	100.0	100.0	100.0	100.0	100.0	100.0	100.0	100.0	100.0
ResNet_v1_152	100.0	100.0	100.0	100.0	100.0	100.0	100.0	100.0	100.0	100.0	100.0	100.0
ResNet_v2_50	100.0	92.3	96.0	100.0	100.0	100.0	100.0	100.0	100.0	100.0	84.6	92.0
ResNet_v2_101	100.0	100.0	100.0	100.0	100.0	100.0	100.0	100.0	100.0	100.0	84.6	92.0
ResNet_v2_152	100.0	92.3	96.0	100.0	100.0	100.0	100.0	100.0	100.0	100.0	100.0	100.0
VGGNet_16	100.0	100.0	100.0	100.0	92.3	96.0	100.0	100.0	100.0	100.0	100.0	100.0
VGGNet_19	100.0	100.0	100.0	100.0	100.0	100.0	100.0	100.0	100.0	100.0	100.0	100.0

**Table 2:** Accuracy (%) results of each model for predicting normal, superficial, and deep.

Models	Raw images				Flipped images				Rotated images				Flipped and rotated images			
	Normal	Superficial	Deep	Total	Normal	Superficial	Deep	Total	Normal	Superficial	Deep	Total	Normal	Superficial	Deep	Total
Inception_v1	66.7	100.0	84.6	85.3	88.9	100.0	92.3	94.1	66.7	100.0	76.9	82.4	88.9	100.0	92.3	94.1
Inception_v2	66.7	83.3	84.6	79.4	100.0	100.0	61.5	85.3	33.3	66.7	61.5	55.9	55.6	91.7	84.6	79.4
Inception_v3	77.8	100.0	84.6	88.2	88.9	100.0	92.3	94.1	66.7	75.0	76.9	73.5	77.8	100.0	69.2	82.4
Inception_v4	77.8	100.0	84.6	88.2	100.0	100.0	92.3	97.1	66.7	100.0	76.9	82.4	77.8	100.0	92.3	91.2
ResNet_v1_50	100.0	100.0	100.0	100.0	100.0	100.0	100.0	100.0	100.0	100.0	100.0	100.0	88.9	100.0	92.3	94.1
ResNet_v1_101	100.0	100.0	100.0	100.0	100.0	100.0	100.0	100.0	66.7	100.0	100.0	91.2	100.0	100.0	100.0	100.0
ResNet_v1_152	88.9	100.0	100.0	97.1	88.9	91.7	92.3	91.2	100.0	100.0	92.3	97.1	100.0	100.0	92.3	97.1
ResNet_v2_50	100.0	100.0	100.0	100.0	100.0	100.0	100.0	100.0	77.8	100.0	100.0	94.1	100.0	91.7	92.3	94.1
ResNet_v2_101	88.9	100.0	100.0	97.1	88.9	100.0	100.0	97.1	55.6	91.7	92.3	82.4	100.0	100.0	84.6	94.1
ResNet_v2_152	100.0	100.0	100.0	100.0	88.9	100.0	100.0	97.1	88.9	83.3	84.6	85.3	88.9	100.0	92.3	94.1
VGGNet_16	88.9	100.0	100.0	97.1	88.9	100.0	100.0	97.1	77.8	100.0	100.0	94.1	77.8	100.0	100.0	94.1
VGGNet_19	88.9	100.0	100.0	97.1	88.9	100.0	100.0	97.1	88.9	100.0	100.0	97.1	88.9	91.7	100.0	94.1

## Discussion

Corneal ulcers are a break in the corneal epithelium that exposes the underlying corneal stroma^12^. Uncomplicated superficial ulcers usually heal rapidly, with minimal scar formation; however, complicated deep ulcers may lead to impaired vision due to corneal scarring or when corneal perforation occurs to anterior synechiae formation^12^. Stromal defects should be cultured, and corneal scrapings for cytological examination should be conducted to determine the underlying etiology due to the high likelihood of microbial infection^12^. Therefore, the distinction between superficial and deep corneal ulcers leads to different treatment results for corneal ulcers. In addition, many deep corneal ulcers require surgical intervention’ consequently, distinguishing the ulcer type is important. Typically, general veterinary surgeons do not find it easy to distinguish between superficial and deep corneal ulcers, which can lead to poor clinical results. In the clinic, any assistance with classifying corneal ulcers may be helpful for the treatment or referral of clinicians.

**Figure 2:**
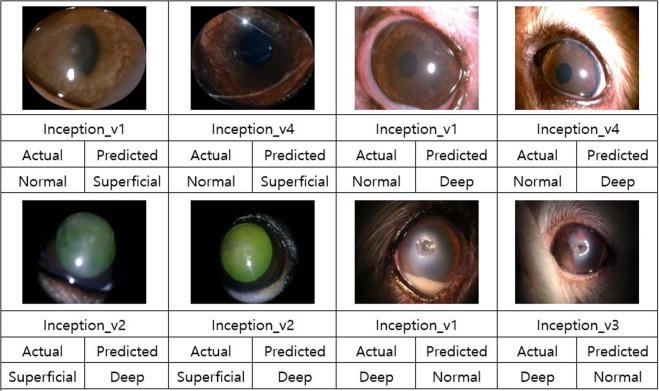
Examples of inaccurately classified images.

In this study, discrimination of corneal ulcer severity in dogs was performed using CNN models. Most models exhibited 100% accuracy in classifying superficial and deep corneal ulcers, and ResNet and VGGNet achieved an accuracies of over 90% for classifying normal corneas, corneas with superficial ulcers, and corneas with deep ulcers, while ResNet_v1_50, ResNet_v1_101, and ResNet_v1_152 mostly achieved accuracies of 90%. ResNet, one of the most recent CNN models, won the ILSVRC in 2015 with its excellent performance. A deeper network will generally perform better, although networks that exceed a certain depth threshold will exhibit larger errors and poor learning effects. ResNet can learn according to its depth and its residual learning approach means it can be used to create extremely deep networks with over 100 layers. Residual learning is a method of learning small variations in the input by finding the differences between input and output by altering the viewpoint of the existing CNN model that finds output values. This approach effectively reduces the number of computations and simplifies the hierarchy by creating direct shortcut connections between the input and the output. Among the tested models, GoogLeNet achieved the worst results. The images in this study were collected under different conditions (exhibiting differences in brightness and contrast) from examination rooms at 4 different veterinary ophthalmology hospitals. The performance of GoogLeNet is affected by brightness and contrast^17^. Figure 2 shows some examples of inaccurately classified images (See supplementary information for more detailed results).

This study proposed a method for effectively discriminating corneal ulcer severity in dogs using a CNN, which is a recently developed deep-learning-based image classification method. A CNN applies convolutional filters to the image to speed computation, and it can be characterized as excellent in that it can process not only a single specific object but also the entire image, including the background. Thus, there have recently been various attempts at CNN-based image recognition and classification. CNNs have been used for disease discrimination in human medical fields, for example, CNNs are used by dermatologists to visually detect skin cancer^18^. CNN-based methods produce better results than traditional methods for tumor classification^17^. As another example, a CNN can distinguish normal optical coherence tomography images from those from patients with age-related macular degeneration^19,20^. We concluded that it is possible to determine corneal ulcer severity via empirical analysis using various CNN models. In this study, we developed a severity classification model was developed that reflects the clinical and epidemiological characteristics of existing corneal ulcers in dogs rather than ulcers evaluated during postoperative follow-up, confirming that deep learning can help diagnose the severity of corneal ulcers.

An important limitation of this study is that we classified the corneal ulcers only by the depth of the corneal involvement. However, corneal ulcers are affected not only by the depth of corneal involvement but also by the presence of bacteria and viruses, the severity of inflammation, and the condition of the cornea and other tissues. However, considering the number of cases in all these categories, it is impossible to accommodate all of them because the number of cases is too large. Therefore, we divided the cases into categories based only on the depth of the corneal ulcer. Another important limitation is that we did not attempt to classify descemetoceles and corneal perforations. Because descemetoceles require fluorescein-stained images, we did not consider them. Furthermore, perforations are obvious in the images. Therefore, we simply classified both descemetoceles and perforations as deep corneal ulcers. Another limitation is that the collected pictures of the corneal ulcers were not always taken in a constant environment, which made it difficult to determine the images suitable for use by the deep learning models. Because of the different conditions in which the pictures were taken, we cannot attribute the effects of the results to deep learning.

## Methods

A total of 368 corneal images were obtained from corneal ulcer patients who visited the Veterinary Medical Teaching Hospital (VMTH) at Konkuk University from 2015 to 2017. The remaining 158 corneal images were collected from three different veterinary ophthalmology specialty hospitals. All methods carried out in accordance with Konkuk University’s animal welfare guidelines. The VMTH, KonKuk University, as a routine procedure, requests the owners of all the animals enrolled in the study, to fill out a patient consent form, which includes the notification that, patient information obtained during treatment may be used for research purposes. We collected information only from those patients whose owners submitted the completed consent form. The other three veterinary ophthalmology specialty hospitals also obtained consent from the owners in the same manner prior to collecting images and information pertaining to the study.

**Figure 3:**
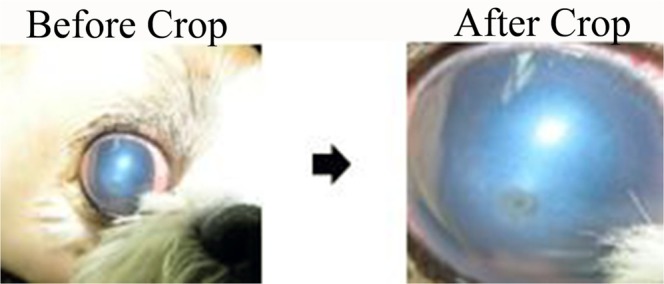
Example of the cropping performed prior to image processing.

A portion of the collected data was not suitable for model learning; therefore, the images were cropped to include only the lesion, as shown in Fig. 3. Few images of corneas with normal conditions appeared among the collected images (n = 57); there were larger numbers of images of superficial (n = 273) and deep (n = 196) ulcers. Note that images are predominantly recorded in cases where disease is suspected. Because it is important to balance the numbers of each class when training CNN models, images of superficial or deep ulcers that were irrelevant, unclear, and duplicates were excluded. We also excluded images that failed to cover the entire cornea. If we could not obtain the entire cornea image because of hair, nictitating membrane, or an incompletely open eye, we excluded that image from the experimental image set.

Additionally, the sizes of the images differ; the largest was 5,184 × 3,456 pixels, and the smallest was 1,184 × 831 pixels. The largest and smallest cropped images were 2,572 × 2,672 and 177 × 134 pixels, respectively. The images were resized to 224 × 224 pixels to train the CNN model.

**Figure 4:**
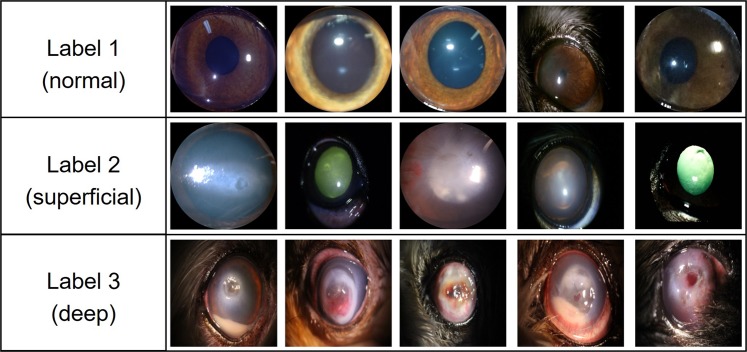
Examples of images with each class label.

Figure 4 describes the labeling. The purpose of this study was to evaluate the severity of corneal ulcers by labeling lesions. A label of “1” (n = 36) denoted the normal condition in which no ulcer was found. A label of “2” (n = 47) indicated a superficial corneal ulcer with damage restricted to the corneal epithelial layer. Deep corneal ulcers with a corneal stromal defect were assigned a label of “3” (n = 47). A lesion was considered a deep corneal ulcer when more than a quarter of the image indicated suspected stromal damage, descemetoceles or corneal perforation. The corneal ulcer severity was classified according to the recording physician’s diagnosis for all the corneal images.

The labeled image data were divided into a training image data set (normal, n = 27; superficial, n = 35; deep, n = 34) to be learned by the model and a validation image data set (normal, n = 9; superficial, n = 12; deep, n = 13) used for accuracy evaluation based on the learned model. To obtain additional images, data augmentation operations such as rotation^21,22^ (normal, n = 144; superficial, n = 188; deep, n = 188), flipping^23^ (normal, n = 144; superficial, n = 188; deep n = 188), and flipping after rotation (normal, n = 288; superficial, n = 376; deep, n = 376) were conducted. Both horizontal and vertical flipping were included; the rotations consisted of 90, 180, and 270 degrees.

In this study, the labeled images were used to train the GoogLeNet, ResNet, and VGGNet models. To accomplish this task, we adopted TensorFlow, an open-source software library developed by Google. The labeled images were converted into TensorFlow record (TFRecord) format for model training and evaluation. A TFRecord is a preprocessed data set that has been transformed to allow it to be recognized by TensorFlow. The experiment was divided into ‘superficial and deep’ and ‘normal, superficial, and deep’ to assess the performances of the models. The models were fine-tuned using the weights of the fully connected layer starting with a CNN model trained by ImageNet and then used to predict severity. For this purpose, we obtained TensorFlow-Slim image classification model code (https://github.com/TensorFlow/models/tree/master/research/slim) from GitHub.

## Conclusions

The results of the empirical analysis in this study indicated that the severity assessments of corneal ulcers by three CNN models exceeded 90% accuracy when classifying superficial and deep corneal ulcers. ResNet and VGGNet achieved an accuracy of over 90% in classifying normal, superficial, and deep corneal ulcers. High accuracy has been achieved due to using the high-quality images in this study. If a significant portion of the image is not actually available or degrades in clinical practice, its usefulness in actual clinical applications may be limited.

The limitations of this study lie mainly with the manual image cropping operations. Model performance can be highly dependent on how the images are cropped. One promising future project would be to train another neural network to automatically locate the eye in the image, which should be a highly accurate task based on recent computer vision literature. Such an approach would make the process more useful and accessible. Additionally, we expect that implementing more detailed models would improve the accuracy on corneal ulcer image data and cause commercialization to become viable as corneal ulcer image data for dogs are Supplemented with the ongoing help of professional medical institutions. In addition, the models tested here are expected to be applicable to other diseases, including human corneal diseases, via discrimination studies of corneal ulcer severity in dogs.

## Supplementary information

Supplementary Information
