# Supplementary material for: CNN-based diagnosis models for canine ulcerative keratitis
Source: Sci Rep. 2019 Oct 2;9:14209. doi: 10.1038/s41598-019-50437-0 (PMC6775068; doi:10.1038/s41598-019-50437-0)
Supplement: Supplementary file 1 — Supplementary Information [file 41598_2019_50437_MOESM1_ESM.pdf]

Article type: Original article

CNN-based diagnosis models for canine ulcerative keratitis

Running head: Classifying canine ulcerative keratitis

Joon Young Kim[1], Ha Eun Lee[1], Yeon Hyung Choi[1], Suk Jun Lee[2,*] & Jong Soo Jeon[2,*]

[1]Veterinary Medical Teaching Hospital, Konkuk University, Seoul, 05029, Republic of Korea

[2]Division of Business Administration, College of Business, Kwangwoon University, Seoul, 01897, Republic of Korea

* Correspondence and requests for materials should be addressed to S.J.L. (email: sjlee@kw.ac.kr) and J.S.J. (email: qws0405@naver.com)

1. The confusion matrix for superficial and deep labels(Raw images).

		actual				actual				actual				actual	
	inception_v1	Superficial	Deep		inception_v2	Superficial	Deep		inception_v3	Superficial	Deep		inception_v4	Superficial	Deep
predict	Superficial	12	0	predict	Superficial	12	3	predict	Superficial	12	1	predict	Superficial	12	0
	Deep	0	13		Deep	0	10		Deep	0	12		Deep	0	13
		actual				actual				actual					
	resnet_v1_50	Superficial	Deep		resnet_v1_101	Superficial	Deep		resnet_v1_152	2	3				
predict	Superficial	12	0	predict	Superficial	12	0	predict	Superficial	12	0				
	Deep	0	13		Deep	0	13		Deep	0	13				
		actual				actual				actual					
	resnet_v2_50	Superficial	Deep		resnet_v2_101	Superficial	Deep		resnet_v2_152	Superficial	Deep				
predict	Superficial	12	1	predict	Superficial	12	0	predict	Superficial	12	1				
	Deep	0	12		Deep	0	13		Deep	0	12				
		actual				actual									
	vgg_16	Superficial	Deep		vgg_19	Superficial	Deep								
predict	Superficial	12	0	predict	Superficial	12	0								
	Deep	0	13		Deep	0	13								

2. The confusion matrix for superficial and deep labels(Flipping images).

		actual				actual				actual				actual	
	inception_v1	Superficial	Deep		inception_v2	Superficial	Deep		inception_v3	Superficial	Deep		inception_v4	Superficial	Deep
predict	Superficial	12	1	predict	Superficial	12	1	predict	Superficial	12	0	predict	Superficial	12	1
	Deep	0	12		Deep	0	12		Deep	0	13		Deep	0	12
		actual				actual				actual					
	resnet_v1_50	Superficial	Deep		resnet_v1_101	Superficial	Deep		resnet_v1_152	Superficial	Deep				
predict	Superficial	12	0	predict	Superficial	12	0	predict	Superficial	12	0				
	Deep	0	13		Deep	0	13		Deep	0	13				
		actual				actual				actual					
	resnet_v2_50	Superficial	Deep		resnet_v2_101	Superficial	Deep		resnet_v2_152	Superficial	Deep				
predict	Superficial	12	0	predict	Superficial	12	0	predict	Superficial	12	0				
	Deep	0	13		Deep	0	13		Deep	0	13				
		actual				actual									
	vgg_16	Superficial	Deep		vgg_19	Superficial	Deep								
predict	Superficial	12	1	predict	Superficial	12	0								
	Deep	0	12		Deep	0	13								

3. The confusion matrix for superficial and deep labels(Rotation images).

		actual				actual				actual				actual	
	inception_v1	Superficial	Deep		inception_v2	Superficial	Deep		inception_v3	Superficial	Deep		inception_v4	Superficial	Deep
predict	Superficial	12	1	predict	Superficial	12	3	predict	Superficial	12	1	predict	Superficial	12	2
	Deep	0	12		Deep	0	10		Deep	0	12		Deep	0	11
		actual				actual				actual					
	resnet_v1_50	Superficial	Deep		resnet_v1_101	Superficial	Deep		resnet_v1_152	Superficial	Deep				
predict	Superficial	12	0	predict	Superficial	12	0	predict	Superficial	12	0				
	Deep	0	13		Deep	0	13		Deep	0	13				
		actual				actual				actual					
	resnet_v2_50	Superficial	Deep		resnet_v2_101	Superficial	Deep		resnet_v2_152	Superficial	Deep				
predict	Superficial	12	0	predict	Superficial	12	0	predict	Superficial	12	0				
	Deep	0	13		Deep	0	13		Deep	0	13				
		actual				actual									
	vgg_16	Superficial	Deep		vgg_19	Superficial	Deep								
predict	Superficial	12	0	predict	Superficial	12	0								
	Deep	0	13		Deep	0	13								

4. The confusion matrix for superficial and deep labels(Flipping & Rotation images).

		actual				actual				actual				actual	
	inception_v1	Superficial	Deep		inception_v2	Superficial	Deep		inception_v3	Superficial	Deep		inception_v4	Superficial	Deep
predict	Superficial	12	2	predict	Superficial	11	2	predict	Superficial	11	2	predict	Superficial	12	3
	Deep	0	11		Deep	1	11		Deep	1	11		Deep	0	10
		actual				actual				actual					
	resnet_v1_50	Superficial	Deep		resnet_v1_101	Superficial	Deep		resnet_v1_152	Superficial	Deep				
predict	Superficial	12	0	predict	Superficial	12	0	predict	Superficial	12	0				
	Deep	0	13		Deep	0	13		Deep	0	13				
		actual				actual				actual					
	resnet_v2_50	Superficial	Deep		resnet_v2_101	Superficial	Deep		resnet_v2_152	Superficial	Deep				
predict	Superficial	12	2	predict	Superficial	12	2	predict	Superficial	12	0				
	Deep	0	11		Deep	0	11		Deep	0	13				
		actual				actual									
	vgg_16	Superficial	Deep		vgg_19	Superficial	Deep								
predict	Superficial	12	0	predict	Superficial	12	0								
	Deep	0	13		Deep	0	13								

5. The accuracy of superficial and deep labels.

Models	Accuracy(%)											
	Raw			Flipping			Rotation			Flipping & Rotation		
	Superficial	Deep	Total	Superficial	Deep	Total	Superficial	Deep	Total	Superficial	Deep	Total
Inception_v1	100.0%	100.0%	100.0%	100.0%	92.3%	96.0%	100.0%	92.3%	96.0%	100.0%	84.6%	92.0%
Inception_v2	100.0%	76.9%	88.0%	100.0%	92.3%	96.0%	100.0%	76.9%	88.0%	91.7%	84.6%	88.0%
Inception_v3	100.0%	92.3%	96.0%	100.0%	100.0%	100.0%	100.0%	92.3%	96.0%	91.7%	84.6%	88.0%
Inception_v4	100.0%	100.0%	100.0%	100.0%	92.3%	96.0%	100.0%	84.6%	92.0%	100.0%	76.9%	88.0%
ResNet_v1_50	100.0%	100.0%	100.0%	100.0%	100.0%	100.0%	100.0%	100.0%	100.0%	100.0%	100.0%	100.0%
ResNet_v1_101	100.0%	100.0%	100.0%	100.0%	100.0%	100.0%	100.0%	100.0%	100.0%	100.0%	100.0%	100.0%
ResNet_v1_152	100.0%	100.0%	100.0%	100.0%	100.0%	100.0%	100.0%	100.0%	100.0%	100.0%	100.0%	100.0%
ResNet_v2_50	100.0%	92.3%	96.0%	100.0%	100.0%	100.0%	100.0%	100.0%	100.0%	100.0%	84.6%	92.0%
ResNet_v2_101	100.0%	100.0%	100.0%	100.0%	100.0%	100.0%	100.0%	100.0%	100.0%	100.0%	84.6%	92.0%
ResNet_v2_152	100.0%	92.3%	96.0%	100.0%	100.0%	100.0%	100.0%	100.0%	100.0%	100.0%	100.0%	100.0%
VGGNet_16	100.0%	100.0%	100.0%	100.0%	92.3%	96.0%	100.0%	100.0%	100.0%	100.0%	100.0%	100.0%
VGGNet_19	100.0%	100.0%	100.0%	100.0%	100.0%	100.0%	100.0%	100.0%	100.0%	100.0%	100.0%	100.0%

6. The confusion matrix for normal, superficial, and deep labels(Raw images).

		actual					actual					actual					actual		
	inception_v1	Normal	Superficial	Deep		inception_v2	Normal	Superficial	Deep		inception_v3	Normal	Superficial	Deep		inception_v4	Normal	Superficial	Deep
	Normal	6	0	1		Normal	6	0	2		Normal	7	0	1		Normal	7	0	0
predict	Superficial	1	12	1	predict	Superficial	1	10	0	predict	Superficial	1	12	1	predict	Superficial	0	12	2
	Deep	2	0	11		Deep	2	2	11		Deep	1	0	11		Deep	2	0	11
		actual					actual					actual							
	resnet_v1_50	Normal	Superficial	Deep		resnet_v1_101	Normal	Superficial	Deep		resnet_v1_152	Normal	Superficial	Deep					
	Normal	9	0	0		Normal	9	0	0		Normal	8	0	0					
predict	Superficial	0	12	0	predict	Superficial	0	12	0	predict	Superficial	0	12	0					
	Deep	0	0	13		Deep	0	0	13		Deep	1	0	13					
		actual					actual					actual							
	resnet_v2_50	Normal	Superficial	Deep		resnet_v2_101	Normal	Superficial	Deep		resnet_v2_152	Normal	Superficial	Deep					
	Normal	9	0	0		Normal	8	0	0		Normal	9	0	0					
predict	Superficial	0	12	0	predict	Superficial	0	12	0	predict	Superficial	0	12	0					
	Deep	0	0	13		Deep	1	0	13		Deep	0	0	13					
		actual					actual												
	vgg_16	Normal	Superficial	Deep		vgg_19	Normal	Superficial	Deep										
	Normal	8	0	0		Normal	8	0	0										
predict	Superficial	0	12	0	predict	Superficial	0	12	0										
	Deep	1	0	13		Deep	1	0	13										

7. The confusion matrix for normal, superficial, and deep labels(Flipping images).

		actual					actual					actual					actual		
	inception_v1	Normal	Superficial	Deep		inception_v2	Normal	Superficial	Deep		inception_v3	Normal	Superficial	Deep		inception_v4	Normal	Superficial	Deep
	Normal	8	0	0		Normal	9	0	2		Normal	8	0	1		Normal	9	0	0
predict	Superficial	0	12	1	predict	Superficial	0	12	3	predict	Superficial	0	12	0	predict	Superficial	0	12	1
	Deep	1	0	12		Deep	0	0	8		Deep	1	0	12		Deep	0	0	12
		actual					actual					actual							
	resnet_v1_50	Normal	Superficial	Deep		resnet_v1_101	Normal	Superficial	Deep		resnet_v1_152	Normal	Superficial	Deep					
	Normal	9	0	0		Normal	9	0	0		Normal	8	0	1					
predict	Superficial	0	12	0	predict	Superficial	0	12	0	predict	Superficial	0	11	0					
	Deep	0	0	13		Deep	0	0	13		Deep	1	1	12					
		actual					actual					actual							
	resnet_v2_50	Normal	Superficial	Deep		resnet_v2_101	Normal	Superficial	Deep		resnet_v2_152	Normal	Superficial	Deep					
	Normal	9	0	0		Normal	8	0	0		Normal	8	0	0					
predict	Superficial	0	12	0	predict	Superficial	0	12	0	predict	Superficial	0	12	0					
	Deep	0	0	13		Deep	1	0	13		Deep	1	0	13					
		actual					actual												
	vgg_16	Normal	Superficial	Deep		vgg_19	Normal	Superficial	Deep										
	Normal	8	0	0		Normal	8	0	0										
predict	Superficial	0	12	0	predict	Superficial	0	12	0										
	Deep	1	0	13		Deep	1	0	13										

8. The confusion matrix for normal, superficial, and deep labels(Rotation images).

		actual					actual					actual					actual		
	inception_v1	Normal	Superficial	Deep		inception_v2	Normal	Superficial	Deep		inception_v3	Normal	Superficial	Deep		inception_v4	Normal	Superficial	Deep
	Normal	6	0	2		Normal	3	1	1		Normal	6	1	1		Normal	6	0	1
predict	Superficial	0	12	1	predict	Superficial	5	8	4	predict	Superficial	0	9	2	predict	Superficial	0	12	2
	Deep	3	0	10		Deep	1	3	8		Deep	3	2	10		Deep	3	0	10
		actual					actual					actual							
	resnet_v1_50	Normal	Superficial	Deep		resnet_v1_101	Normal	Superficial	Deep		resnet_v1_152	Normal	Superficial	Deep					
	Normal	9	0	0		Normal	6	0	0		Normal	9	0	1					
predict	Superficial	0	12	0	predict	Superficial	0	12	0	predict	Superficial	0	12	0					
	Deep	0	0	13		Deep	3	0	13		Deep	0	0	12					
		actual					actual					actual							
	resnet_v2_50	Normal	Superficial	Deep		resnet_v2_101	Normal	Superficial	Deep		resnet_v2_152	Normal	Superficial	Deep					
	Normal	7	0	0		Normal	5	0	1		Normal	8	2	1					
predict	Superficial	0	12	0	predict	Superficial	1	11	0	predict	Superficial	1	10	1					
	Deep	2	0	13		Deep	3	1	12		Deep	0	0	11					
		actual					actual												
	vgg_16	Normal	Superficial	Deep		vgg_19	Normal	Superficial	Deep										
	Normal	7	0	0		Normal	8	0	0										
predict	Superficial	0	12	0	predict	Superficial	0	12	0										
	Deep	2	0	13		Deep	1	0	13										

9. The confusion matrix for normal, superficial, and deep labels(Flipping & Rotation images).

		actual					actual					actual					actual		
	inception_v1	Normal	Superficial	Deep		inception_v2	Normal	Superficial	Deep		inception_v3	Normal	Superficial	Deep		inception_v4	Normal	Superficial	Deep
	Normal	8	0	0		Normal	5	0	1		Normal	7	0	2		Normal	7	0	0
predict	Superficial	0	12	1	predict	Superficial	1	11	1	predict	Superficial	0	12	2	predict	Superficial	0	12	1
	Deep	1	0	12		Deep	3	1	11		Deep	2	0	9		Deep	2	0	12
		actual					actual					actual							
	resnet_v1_50	Normal	Superficial	Deep		resnet_v1_101	Normal	Superficial	Deep		resnet_v1_152	Normal	Superficial	Deep					
	Normal	8	0	1		Normal	9	0	0		Normal	9	0	1					
predict	Superficial	0	12	0	predict	Superficial	0	12	0	predict	Superficial	0	12	0					
	Deep	1	0	12		Deep	0	0	13		Deep	0	0	12					
		actual					actual					actual							
	resnet_v2_50	Normal	Superficial	Deep		resnet_v2_101	Normal	Superficial	Deep		resnet_v2_152	Normal	Superficial	Deep					
	Normal	9	1	0		Normal	9	0	2		Normal	8	0	0					
predict	Superficial	0	11	1	predict	Superficial	0	12	0	predict	Superficial	0	12	1					
	Deep	0	0	12		Deep	0	0	11		Deep	1	0	12					
		actual					actual												
	vgg_16	Normal	Superficial	Deep		vgg_19	Normal	Superficial	Deep										
	Normal	7	0	0		Normal	8	0	0										
predict	Superficial	0	12	0	predict	Superficial	0	11	0										
	Deep	2	0	13		Deep	1	1	13										

10. The accuracy of superficial and deep labels.

Models	Accuracy(%)															
	Raw				Flipping				Rotation				Flipping & Rotation			
	Normal	Superficial	Deep	Total	Normal	Superficial	Deep	Total	Normal	Superficial	Deep	Total	Normal	Superficial	Deep	Total
Inception_v1	66.7%	100.0%	84.6%	85.3%	88.9%	100.0%	92.3%	94.1%	66.7%	100.0%	76.9%	82.4%	88.9%	100.0%	92.3%	94.1%
Inception_v2	66.7%	83.3%	84.6%	79.4%	100.0%	100.0%	61.5%	85.3%	33.3%	66.7%	61.5%	55.9%	55.6%	91.7%	84.6%	79.4%
Inception_v3	77.8%	100.0%	84.6%	88.2%	88.9%	100.0%	92.3%	94.1%	66.7%	75.0%	76.9%	73.5%	77.8%	100.0%	69.2%	82.4%
Inception_v4	77.8%	100.0%	84.6%	88.2%	100.0%	100.0%	92.3%	97.1%	66.7%	100.0%	76.9%	82.4%	77.8%	100.0%	92.3%	91.2%
ResNet_v1_50	100.0%	100.0%	100.0%	100.0%	100.0%	100.0%	100.0%	100.0%	100.0%	100.0%	100.0%	100.0%	88.9%	100.0%	92.3%	94.1%
ResNet_v1_101	100.0%	100.0%	100.0%	100.0%	100.0%	100.0%	100.0%	100.0%	66.7%	100.0%	100.0%	91.2%	100.0%	100.0%	100.0%	100.0%
ResNet_v1_152	88.9%	100.0%	100.0%	97.1%	88.9%	91.7%	92.3%	91.2%	100.0%	100.0%	92.3%	97.1%	100.0%	100.0%	92.3%	97.1%
ResNet_v2_50	100.0%	100.0%	100.0%	100.0%	100.0%	100.0%	100.0%	100.0%	77.8%	100.0%	100.0%	94.1%	100.0%	91.7%	92.3%	94.1%
ResNet_v2_101	88.9%	100.0%	100.0%	97.1%	88.9%	100.0%	100.0%	97.1%	55.6%	91.7%	92.3%	82.4%	100.0%	100.0%	84.6%	94.1%
ResNet_v2_152	100.0%	100.0%	100.0%	100.0%	88.9%	100.0%	100.0%	97.1%	88.9%	83.3%	84.6%	85.3%	88.9%	100.0%	92.3%	94.1%
VGGNet_16	88.9%	100.0%	100.0%	97.1%	88.9%	100.0%	100.0%	97.1%	77.8%	100.0%	100.0%	94.1%	77.8%	100.0%	100.0%	94.1%
VGGNet_19	88.9%	100.0%	100.0%	97.1%	88.9%	100.0%	100.0%	97.1%	88.9%	100.0%	100.0%	97.1%	88.9%	91.7%	100.0%	94.1%

11. Examples of unclear/irrelevant images

Cropped images	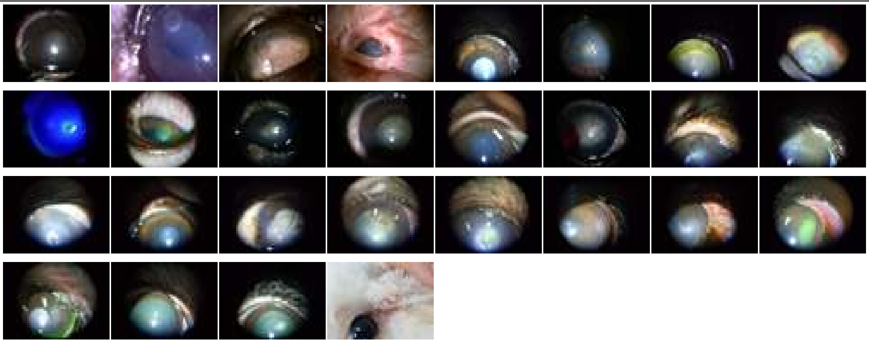
Eyelid and/or NM partially covers the cornea	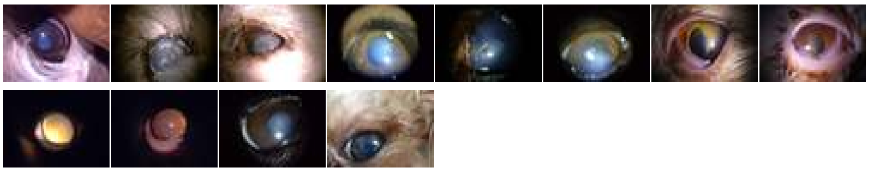
Hair partially covers the cornea	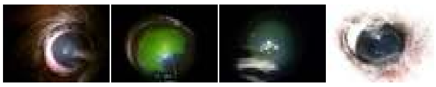
Nasal fold partially covers the cornea	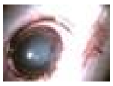
Too dark images	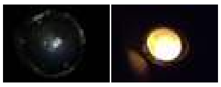
Irrelevant images	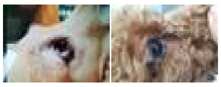
